# Strategic Management Advanced Service for Sustainable Computing Environment

**DOI:** 10.1155/2015/715967

**Published:** 2015-03-30

**Authors:** Sang-Soo Yeo, Qun Jin, Vincenzo Loia, Hangbae Chang

**Affiliations:** ^1^Division of Convergence Computer & Media, Mokwon University, Daejeon 302-729, Republic of Korea; ^2^Department of Human Informatics and Cognitive Sciences, Waseda University, Tokorozawa 359-1192, Japan; ^3^Department of Mathematics and Computer Science, University of Salerno, Fisciano, Salerno, Italy; ^4^Department of Industrial Security, College of Business and Economics, Chung-Ang University, Seoul 156-756, Republic of Korea

Unquestionably, business and industrial information can be considered an extremely important asset to any organization. Some would even go as far as claiming that organization's information resources are the lifeblood of that organization. However, recently other competitors such as countries and enterprises are doing their best to have advanced technology of certain corporation. This business and industrial information leakage tend to halt the ordinary business process of enterprise, causing tremendous economical property loss as well as damage to the competitiveness of enterprise due to the leakage of technology which needs to be effort- and time-consuming.

Only security technology cannot directly protect against the underlying security incidents (business and industrial information leakage) that, in practice, lead to loss. Recent surveys suggest up to 60% of security breaches are related to human problem; yet few companies focus on human aspects in their security strategies. Hence to take preventive measures against security breaches, it is necessary to manage and converge three security areas (managerial, physical, and technical management) in the perspective of corporate and business security strategy.

The main motivation for this special issue is to bring together researchers and practitioners working on related fields in human centric security management and its services to present current research issues and advances. Papers on practical as well as on theoretical topics and problems are invited.

More specifically, the paper entitled “A Comprehensive Availability Modeling and Analysis of a Virtualized Servers System Using Stochastic Reward Nets” by T. A. Nguyen et al. proposed a virtualized servers system with multiple VMs via SRN. This paper encapsulated four VMs running on two VMMs into two hosts, and it also incorporated diverse failure modes and corresponding recovery behaviors regarding hardware and software aspects including host failure, SAN failure, aging-related failure, and Mandelbugs related failure in SRN models. The paper entitled “Software Authority Transition through Multiple Distributors” by K. Han and T. Shon discussed possible issues from using multiple OASs and proposed an improved PAS model that reduces management overheads without any additional entity, while still allowing users to obtain support from multiple OASs. This paper refined our model to support a temporary roaming situation, as well as a permanent OAS change. And it described the security of the proposed model. The paper entitled “The Study on Stage Financing Model of IT Project Investment” by S. Chen et al. applies the real option pricing model to measure the value brought by the stage financing strategy. The paper entitled “Advanced Approach to Information Security Management System Model for Industrial Control System” by S. Park and K. Lee presented two methodologies to prove that a new information security management system based on confidentiality, integrity, availability, and safety is required in the industrial control system. The paper entitled “An Integrative Behavioral Model of Information Security Policy Compliance” by S. H. Kim et al. tried to find the factors of information security policy compliance and suggest the information security policy based upon the founded factors. The paper entitled “The Strategic Measures for the Industrial Security of Small and Medium Business” by C.-M. Lee presented that online security control services and technology deposit system are suggested for such measures. These measures could enhance to a certain extent the industrial security of SMB. The low security awareness and financial difficulties seem to be the main obstacles to equip the SMB with such measures. The paper entitled “AVQS: Attack Route-Based Vulnerability Quantification Scheme for Smart Grid” by J. Ko et al. proposed a novel AVQS to accurately measure the security level in a smart grid. The proposed approach includes NVS and end-to-end security functions. The paper entitled “The Need for Specific Penalties for Hacking in Criminal Law” by S. Oh and K. Lee analyzed the definitions and the penalties for hacking for each country and compared with the national law; then it made suggestions through more specific legislation. This paper expects it will reduce legal controversy and prevent excessive punishment. The paper entitled “Empirical Analysis of Retirement Pension and IFRS Adoption Effects on Accounting Information: Glance at IT Industry” by J. Kim reviewed new pension accounting with K-IFRS and provided empirical changes in liability for retirement allowances with adoption of K-IFRS. It will help to understand the effect of pension accounting on individual firm's financial report and the importance of public announcement of actuarial assumptions. The paper entitled “Security Techniques for Prevention of Rank Manipulation in Social Tagging Services including Robotic Domains” by O. Choi et al. proposed a detection method for tag-ranking manipulation to solve the problem of the existing methods which cannot guarantee the reliability of tagging. The paper entitled “Effects of Corporate Social Responsibility and Governance on Its Credit Ratings” by D. Kim and J. Kim showed nonfinancial information also may have effects on corporate credit rating. The investment on personal data protection could be an example of CSR/CGI activities which have positive effects on corporate credit ratings.

Eventually, we firmly believe that the accepted papers would be a meaningful contribution to researchers, students, and practitioners studying this field of strategic management advanced service for sustainable computing environment.

